# Microgreens Biometric and Fluorescence Response to Iron (Fe) Biofortification

**DOI:** 10.3390/ijms232314553

**Published:** 2022-11-22

**Authors:** Barbara Frąszczak, Tomasz Kleiber

**Affiliations:** 1 Department of Vegetable Crops, Poznań University of Life Sciences, 60-594 Poznan, Poland; 2 Laboratory of Plant Nutrition, Department of Plant Physiology, Poznań University of Life Sciences, 60-198 Poznan, Poland

**Keywords:** functional food, microscale vegetables, artificial light, microelements

## Abstract

Microgreens are foods with high nutritional value, which can be further enhanced with biofortification. Crop biofortification involves increasing the accumulation of target nutrients in edible plant tissues through fertilization or other factors. The purpose of the present study was to evaluate the potential for biofortification of some vegetable microgreens through iron (Fe) enrichment. The effect of nutrient solution supplemented with iron chelate (1.5, 3.0 mg/L) on the plant’s growth and mineral concentration of purple kohlrabi, radish, pea, and spinach microgreens was studied. Increasing the concentration of Fe in the medium increased the Fe content in the leaves of the species under study, except for radish. Significant interactions were observed between Fe and other microelements (Mn, Zn, and Cu) content in the shoots. With the increase in the intensity of supplementation with Fe, regardless of the species, the uptake of zinc and copper decreased. However, the species examined suggested that the response to Fe enrichment was species-specific. The application of Fe didn’t influence plant height or fresh and dry weight. The chlorophyll content index (CCI) was different among species. With increasing fertilisation intensity, a reduction in CCI only in peas resulted. A higher dose of iron in the medium increased the fluorescence yield of spinach and pea microgreens. In conclusion, the tested species, especially spinach and pea, grown in soilless systems are good targets to produce high-quality Fe biofortified microgreens.

## 1. Introduction

If a person’s diet is varied and balanced and the level of minerals is correct, there is no need to supplement the food. However, in the case of some components (calcium, iron, iodine), deficiencies are quite common, especially in certain physiological conditions, such as pregnancy or lactation [1]. About 800 million people are undernourished worldwide, while between 1.5 to 2 billion people have one or more chronic micronutrient deficiencies, notably deficiencies in the minerals calcium, iodine, iron, selenium, zinc, and vitamins such as folate and vitamin A [2,3]. In Africa and South East Asia, as high as two-thirds of children under five and nearly half of the women suffer from anaemia [4]. For example, in China, the estimated population affected by anemia was 245 million, and 208 million of these were due to iron deficiency. Three-quarters of these affected populations were found in rural areas [5].

Iron (Fe) is a key component of proteins such as haemoglobin and myoglobin that transport oxygen in the human body. It is a component and activator of many enzymes involved in electron transport, oxidation-reduction reactions, and other biological functions [6]. Iron deficiency causes anemia, the most common and widespread nutritional disorder globally [7]. Micronutrient fortification of food is the most practical way of preventing nutrient deficiency in people [8]. Biofortification of rice and wheat plants by foliar spray of iron was an effective way to promote iron concentration in rice and wheat grains [9,10].

Microgreens are more and more important in health-promoting diets. They are considered a valuable source of nutrients and bioactive ingredients, and show potential in the prevention of malnutrition and chronic diseases. Some procedures implemented at the pre- or post-harvest stages of microgreens may be useful in improving the nutritional and functional values of that type of food [11]. One such process is biofortification. Microgreens, like vegetables, can be biofortified with various elements (e.g., iron, selenium, silicon, or iodine) by increasing their concentration in the medium or spraying them with compounds, thus providing missing minerals in the human diet [12]. Previous studies show good absorption of that element from biofortified plants, which may be due to the high content of ascorbic acid in the plants, among other things [13].

Although Fe is the fourth most abundant element in the Earth’s crust, it is poorly bioavailable in the soil as it rapidly binds to soil particles and ions to create forms insoluble under aerobic conditions at neutral or alkaline pH [14]. Its content in the soil is also significantly higher than that of the other micronutrients. Compared to macronutrients, the demand of plants for Fe is relatively low, although it is clearly higher than the demand for other micronutrients. Fe plays a key role in plant growth and productivity. For example, it is a component of many vital enzymes, such as cytochromes, present in the electron transport chain. Thus, it is necessary for a wide range of biological functions. In plants, iron is involved in the synthesis of chlorophyll, and it is essential for the maintenance of chloroplast structure and functioning [15]. An insufficient level of Fe slows down the process of plant growth and has a negative impact on the quality of the crop [16], and, in the case of a longer-lasting deficit, it leads to the so-called chlorosis. In recent years, agronomic biofortification has been more and more often proposed to increase the accumulation of target nutrients in tissues of edible plants through fertilisation or other factors [17,18]. However, large-scale agronomic biofortification involves some risks. Constant supplementary fertilisation of soil with metals, such as iron or zinc, can pose environmental and health risks due to their potential leaching into groundwater or accumulation in soil or plant tissues at excessive levels, which can be toxic to plants and other organisms as well as to the consumer [19,20,21]. Excessive content of some micronutrients can also contribute to antagonism, e.g., between iron and other metal micronutrients (Mn, Zn, Cu, Ni). Therefore, proper control of the concentration of micronutrients and constant exposure of roots to a medium enriched in nutrients, with no soil, can maximise their uptake, transport, and accumulation in edible parts of plants [22] and help to avoid or minimise some of the risks associated with the agronomic biofortification of crops grown in soil conditions. However, it is not easy to enrich plants with the necessary micronutrients without having negative effects, i.e., reduced growth and yield [23].

Microgreens are considered a functional food with high concentrations of mineral nutrients and phytochemicals that contribute to health benefits. They produced 4–40 times the carotenoid concentrations and essential vitamins as a mature plant would [24].

Pea shoots and radish leaves, apart from sunflower seeds, are the most popular microgreens grown in the United States. This is determined by several factors: ease and short cultivation period, low cost of seeds compared to other types of species, high quality of the plants, and good weight efficiency per unit area of the tray [25]. The above-mentioned species are also important in cancer prevention, thanks to their great antiproliferative effects manifested by significantly reducing the spheroid surface area [26]. Spinach, in turn, is a rich source of vitamins, minerals, and trace elements, as well as various bioactive compounds, including carotenoids, flavonoids, and tocopherols [27]. Based on earlier studies, spinach microgreens contain much greater amounts of carotenoids, vitamins [28], or α-tocopherol, and contain less oxalic acid than mature leaves [29]. Moreover, based on the research conducted by Ghoor et al. [29], spinach microgreens have a 2.5–3.0 times higher Nutritional Quality Score (NQS) than mature spinach, which further highlights their importance in a healthy diet.

Kohlrabi, like other *Brassicaceae* microgreens, is characterised by a high content of phytochemicals, vitamins, and mineral elements [30]. The content of flavonoids and glucosinolates determines their antioxidant properties and characteristic smell and taste [31]. Also, kohlrabi microgreens are characterised by high absorption of bioactive compounds [32].

The purpose of the research was to preliminarily assess the utility of the above-mentioned species of microgreens, grown in mineral wool and without soil, for the application of iron biofortification.

## 2. Results

### 2.1. Growth Parameters

**Figure 1:**
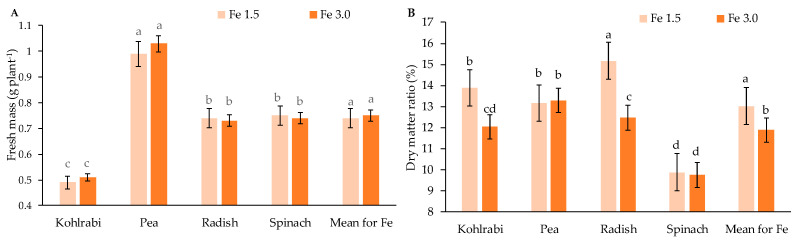
The fresh mass (**A**, g plant^−1^) and dry matter ratio (**B**, %) for four microgreen crops depend on the Fe level. Two levels of iron nutrition in the medium were used: 1.5 and 3.0 mg/L. Different letters for the same parameter indicate significant differences at the 5% level, according to Duncan’s test. The bars represent the standard deviation.

The applied iron (Fe) doses did not significantly differentiate the fresh weight of the microgreens (Figure 1A). However, significant differences were found between species. The highest fresh weight was obtained for pea microgreens, while the lowest was for kohlrabi. The percentage share of dry matter decreased significantly for the Fe 3.0 dose in kohlrabi and radish (Figure 1B). For Fe 1.5, the highest dry matter content was noticed for radish, while the lowest was obtained for spinach.

Differences in biomass production were demonstrated in Table 1—the highest was observed for peas and the lowest for kohlrabi. In the case of kohlrabi and radish, there was a trend, statistically proven, of a significant reduction in the biomass yield with increasing intensity of iron supplementation.

**Figure 2:**
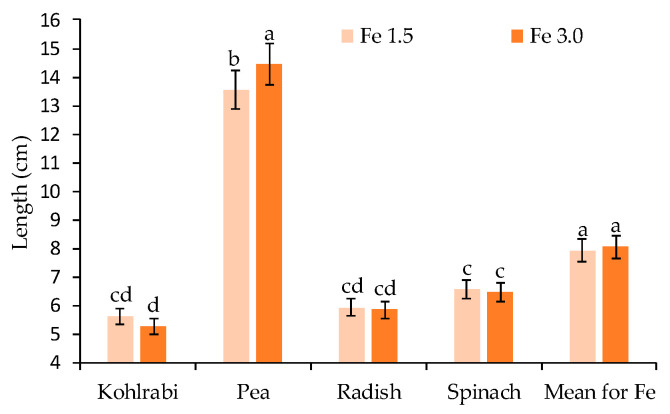
The length (cm) for four microgreen crops depends on the Fe level. Two levels of iron nutrition in the medium were used: 1.5 and 3.0 mg/L. Different letters for the same parameter indicate significant differences at the 5% level, according to Duncan’s test. The bars represent the standard deviation.

The applied Fe doses did not differentiate the plant length, except for pea microgreens, which were significantly longer when supplemented with Fe 3.0 (Figure 2). In the case of that species, their microgreens were the longest among all studied plants.

**Figure 3:**
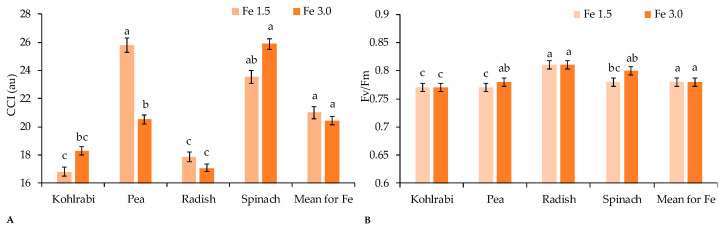
The CCI (**A**) and the fluorescence yield ((**B**), Fv/Fm) for four microgreen crops depend on the Fe level. Two levels of iron nutrition in the medium were used: 1.5 and 3.0 mg/L. Different letters for the same parameter indicate significant differences at the 5% level, according to Duncan’s test. The bars represent the standard deviation.

The chlorophyll content index (CCI) was different and ranged from 16.8 (kohlrabi, Fe 1.5) to 25.8 (spinach, Fe 3.0). The Fe supplementation level influenced the CCI value (Figure 3A). With increasing fertilisation intensity, a 20% reduction in the relative chlorophyll content in peas resulted. In turn, the opposite was observed for spinach and kohlrabi. Fertilizing the plants with the medium that contained Fe 3.0 increased the CCI value in the leaves of both species (by approximately 10%), although those differences were not statistically significant compared to Fe 1.5. It is worth noting that in the case of peas (for Fe 1.5) and spinach (Fe 3.0), the CCI was significantly higher compared to the other species.

The maximum quantum efficiency of photosystem II photochemistry (Fv/Fm) ranged between 0.77 a 0.81 (Figure 3B). A higher dose of iron in the medium increased the fluorescence yield of spinach and pea microgreens. The highest level of fluorescence was obtained for radish.

### 2.2. Minerals Content

The applied diversified supplementation with iron significantly influenced the content of the studied metal micronutrients in the analysed microgreens (Table 2). In all the species under study, a higher content of copper (Cu) was recorded in plants grown on the medium with a lower content of iron (Fe 1.5)—its increase significantly reduced the content of Cu. The largest decrease, approximately 30%, was observed in spinach microgreens. A higher dose of Fe in the medium significantly increased the % of Fe content in the microgreens. Only in the case of radishes was that content the same for both doses. Pea had the highest Fe content for both doses. As a result of the biofortification, the highest increase in Fe (+36%) was observed in spinach. In turn, taking manganese (Mn) into account, increasing the content of Fe in the medium resulted in increased accumulation of that element in the microgreens. For peas and radishes, considering the tested ranges, no effect of Fe biofortification on the content of Mn was noticed. The greatest differences were obtained for kohlrabi (+34%), which was also characterised by the highest content of Mn in the plant. The opposite reaction was observed for zinc (Zn). Increasing the level of Fe in the medium resulted in a reduction of the content of Zn in the plants. The largest, approximately 10%, and significant differences were found for kohlrabi and pea, not statistically significant for spinach.

Iron biofortification influenced the uptake of micronutrients by the aerial parts of microgreens (Table 3). A positive trend for Fe was demonstrated in spinach, for Mn—in cabbage and spinach. With the increase in the intensity of supplementation with Fe, regardless of the species, the uptake of zinc and copper decreased.

The performed correlation analysis showed linear relationships between the examined features (Table 4); for example, the fresh mass (yield) was positively correlated (0.69) with the content of Fe in plants and its uptake, also taking into account the content of dry matter (0.83). The biomass yield was significantly correlated with iron uptake. In turn, the content of iron, as well as iron uptake, was positively correlated with SPAD values (0.70 and 0.40, respectively).

**Figure 4:**
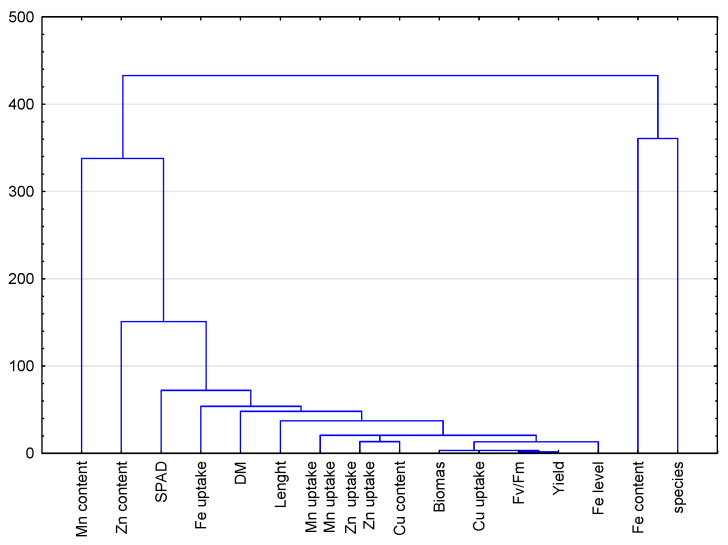
Hierarchical clustering demonstrates studied parameters of microgreens in relation to the level of Fe. Two levels of iron nutrition in the medium were used: 1.5 and 3.0 mg/L.

A cluster analysis was performed by grouping similar features (Figure 4). The tested Fe levels in the medium had the strongest effect on the content of Fe negatively correlated with the content of Mn. Both of those features largely depended on the species of the plant under study.

**Figure 5:**
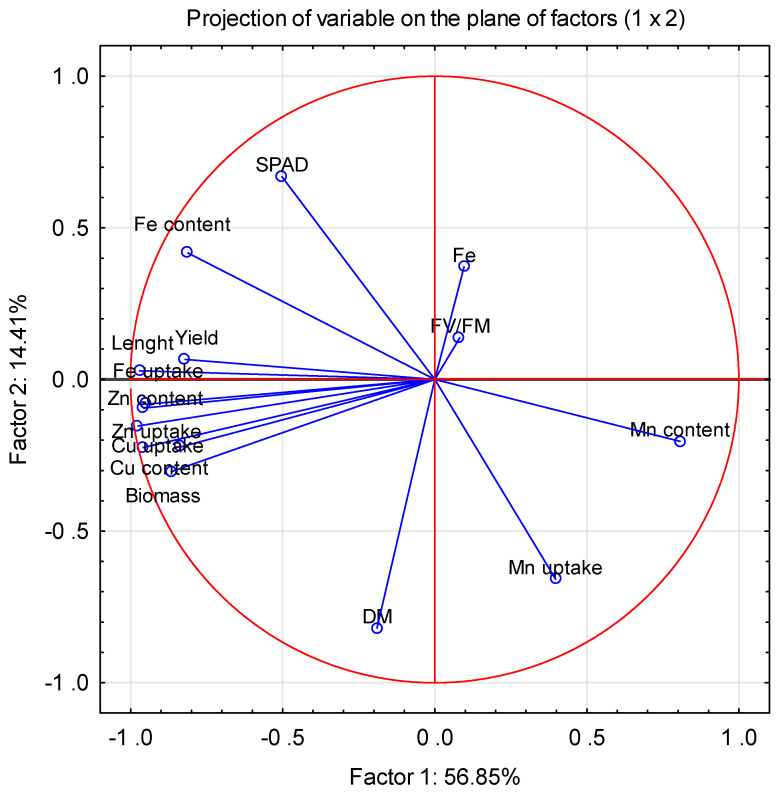
Principal components analysis (PCA) of microgreens showed a correlation between the level of Fe and measured parameters. Two levels of iron nutrition in the medium were used: 1.5 and 3.0 mg/L.

The principal components analysis (PCA) results (Figure 5) showed a positive linear correlation between the length, yield, and biomass of microgreens and the uptake of Fe, Zn, and Cu, as well as the content of Cu and Zn in the plants. A negative correlation was found between SPAD and the uptake and content of Mn and between the level of Fe and Fv/Fm and dry mass ratio (DM). There was no linear correlation between the two main components and the content and uptake of Mn by the plants.

## 3. Discussion

Vegetable biofortification is one of the ways to improve their quality and prevent micronutrient deficiencies in humans. However, it is challenging to increase the production of food enriched with essential micronutrients without having obvious negative symptoms for plants at the same time, such as slower growth and productivity [23]. In the conducted study, no effect of the applied Fe doses on length (Figure 2) and the yield of fresh weight (Figure 2) was noticed, although the yield of biomass declined for species with higher sensitivity (kohlrabi, radish) (Table 1). In the earlier studies by Vaštakaitė-Kairienė et al. [33], different doses of Fe (2, 5, and 15 ppm) in the medium did not significantly affect the biometric parameters of broccoli microgreens. Based on the results obtained in this study, the applied doses did not negatively affect the growth dynamics and did not reduce the fresh mass of the microgreens. Many authors emphasise that a high concentration of Fe in the medium has a toxic effect on plants. In the study by Gioia et al. [34], the greatest obtained yield was in the range of up to 10 mg/L. Higher doses significantly reduced the yield of the tested microgreens. The response of plants to an increase in the level of Fe may vary considerably depending on the species. For example, the enrichment of broccoli and radish sprouts with Fe at concentrations of 24 and 36 mg L^−1^ reduced their yield, while it had no negative effect on the yield of mung bean and alfalfa sprouts [35]. In the present study, the effect of yield reduction due to increased supplementation with Fe was evident for dry matter and biomass (statistically proven for kohlrabi and radish).

The content of dry matter of kohlrabi and radish microgreens was significantly lower for Fe 3.0 dose (Figure 1B). There were no significant differences for the other species, which also shows the different sensitivity of species to Fe concentration in the medium. Although iron is an essential element for the physiological and biochemical processes in plants, an excess of it in plant tissues can contribute to a number of metabolic disorders, mainly due to its involvement in Fenton reactions leading to an increase in the level of reactive oxygen species [36] which can cause oxidative stress in plants.

Iron is essential for the synthesis of chlorophyll [37]. According to previous studies, the biofortification of Fe caused increased chlorophyll content in plants [38]. Based on current research, the increase in the concentration of Fe in the medium only slightly increased CCI in spinach and kohlrabi (by 10 and 9%, respectively, Figure 3A). In contrast, it had a significant effect on the reduction of CCI in peas and a slight reduction of CCI in radishes. Leaving types of microgreens aside, which also have a significant impact on chlorophyll synthesis [39], the lack of a clear positive reaction in plants could have been due to the short growth period and the relatively difficult transport of iron inside the plant. The above-cited authors examined the mature plants after several months of growth, in the case of which the effect of Fe on the synthesis of chlorophyll could be greater compared to microgreens that are two weeks old.

The Fv/Fm ratio can be taken as an indirect indicator of stress. It usually ranges between 0.81 and 0.83 in healthy plants and falls below that level in plants subjected to stress conditions as Fo increases and Fm decreases [40]. However, that value depends on the plant species and environmental conditions [41]. In this study, the Fv/Fm ratio was within the standard range for radish and spinach at Fe 3.0 (Figure 3B). In contrast, for the remaining combinations it was 0.77–0.78, so it was lower than the normal range, which may indicate that the plants were subjected to a certain amount of stress possibly caused by an increased level of Fe. In the available literature, there is no information on the effect of Fe on the maximum quantum yield of PS II. However, it is worth mentioning that in the studies of other authors, the biofortification of various vegetable species with iodine did not affect the Fv/Fm value [42,43]. Based on present research, it can be assumed, taking into account the downward trend in biomass production, that with the increase in the concentration of iron, the potential stress of plants could increase. Also, the phenomena of ion antagonism between Fe and other metal micronutrients can occur. Too much iron interferes with chlorophyll synthesis; pigment disruption of photosynthetic complexes promotes changes in electron transport, which causes a reduction in the net assimilation rate of CO_2_ and deprives the plant of essential sugars [44]. A threefold normal iron level also resulted in abnormal chloroplast structure, reduced net photosynthetic rate, and decreased biomass [45]. This result could be attributed to the high toxicity of iron. The reason for the toxic effect of iron is its reaction with hydrogen peroxide (H_2_O_2_) and hydroxyl radical (OH–), which is the most reactive oxygen species (ROS) [46]. Therefore, it is important to select species capable of accumulating high-affinity iron and to conduct research on the optimisation of plant biofortification with that micronutrient.

Increasing the concentration of Fe in the medium increased the Fe content in the leaves of the species under study, except for radishes (Table 2). Also, in the studies by Przybysz et al. [35], an increase in the content of Fe in radish sprouts was observed only at the highest dose of Fe in the medium (36 mg L ^−1^ Fe). Furthermore, among the three species, spinach was the most sensitive to the Fe level. However, those were not very large increases compared to the results obtained in the studies conducted by other authors, which might have been due to a lower level of Fe in the medium. The uptake of components by plants may depend on their proportions in the medium, inter alia. In his study, De Goia et al. [34] showed a 278% increase in the content of Fe in red cabbage microgreens, with an application of 20 mg L^−1^ Fe, compared to the reference object. In the present research, spinach, characterised by the highest increase in Fe content, did not contain the largest amount of that element. The highest Fe concentrations were found in peas for both combinations. However, there was a slight increase in the Fe content of spinach if there was a higher content of that element in the medium. In the studies by Przybysz et al. [35], radish sprouts were characterised by the highest iron content among the studied species but also by the slowest growth rate of Fe in sprouts with the increase of that element in the medium. This shows that species with a high natural content of Fe in their leaves are not very susceptible to the accumulation of that element with an increased dose of Fe in the medium. Therefore, to achieve the highest possible concentration of Fe in microgreens, it is necessary to efficiently enrich the medium and choose species that are naturally rich in that element and highly sensitive to Fe biofortification.

The conducted study proved a positive correlation between the content of Fe in the medium and the content of Mn in the plants (Table 2). Only for peas was the content the same. This species was also characterised by the lowest content of Mn compared to other microgreens under study. The highest content of Mn was found in the shoots of kohlrabi, which had the lowest content of Fe. In this case, in the analysed concentration range, the antagonism effect between the content of Fe and Mn in the shoots can be indicated. The Fe: Mn ratio in products consumed by people is very important. Those micronutrients “compete” for the same serum protein (transferrin) and transport systems—the DMT 1 (*divalent metal transporter*) [47].

One of the biggest problems related to enriching plants with one ion is the impairment of uptake of others, the so-called antagonism [35]. In the present research, a higher dose of Fe had a negative effect on the content of Cu and Zn in the shoots of microgreens (Table 2). Such a reaction of plants may be caused by, among other things, competition between iron and other cations as they share the same cell membrane transporters [48,49,50]. Moreover, the excess of Fe in the medium causes the formation of the root sheath due to excessive Fe stress, which makes the absorption of components more difficult [51]. Moreover, Souza-Santos et al. [52] showed that Fe causes irreversible inhibition of the activity of H+-ATPase in maize root cell membranes by oxidation of the sulfhydryl groups of the enzyme following lipid peroxidation. In the study by Jalal et al. [53], the mean values of the data revealed that increasing the level of Fe consistently decreased the content of Zn in grain. In contrast, increasing the level of Zn decreased the content of Fe in grain. This is because Fe and Zn have an antagonistic relationship when a certain parallel concentration and Fe transitional phloem mobility is reached [54]. It is worth noting that, in some studies, an increase in the level of Fe in the medium also contributed to an increase in the level of Zn in the leaves [45]. In this research, species differences were also noticeable. In the pea with the highest content of Fe, there was also a high level of Cu and Zn and the lowest level of Mn. In contrast, kohlrabi, with the lowest iron level, contained a high level of Mn and low levels of Zn and Cu. In the case of radishes, the lowest effect of the level of Fe in the media on the content of Fe, Mn, and Zn was found. With regard to uptake, there was a significant difference between the studied species (Table 3). For example, the highest average content of Fe was noticed in peas, and the lowest was in kohlrabi. Similar trends were observed for zinc. Generally, an increased level of supplementation with Fe did not have a positive effect on the uptake of that micronutrient by plants. Among the tested species, spinach was different as there was an upward trend in the intake of the above-mentioned micronutrient by that plant (+16.7%). Similar trends were observed for manganese (+9.9%). As the level of Fe increased, the content of Zn and Cu decreased (on average).

In the soilless cultivation of microgreens, Fe biofortification at the level of 3.0 mg/L can be an effective tool for producing functional foods. However, based on studies by other authors, the range of iron levels suitable for the growth of young seedlings is very narrow [36].

## 4. Materials and Methods

### 4.1. Plant Material and Growth Conditions

Four vegetable species were grown: Purple kohlrabi (*Brassica oleracea* var. *gongylodes)*, radish (*Raphanus sativus* var. *sativus, Brassicaceae*), pea Boogie (*Pisum sativum, Fabaceae*), and spinach (*Spinacia oleracea*, *Chenopodiaceae*). The experiment was conducted in a growth chamber. NEONICA LED 240 (Neonica, Łódź, Poland) modules were used as the light source. The photosynthetic photon flux density (PPFD) was 480 µmol m^−2^ s^−1^, with the following share of individual colours: R (red): 383 (µmol m^−2^ s^−1^); G (green): 33 (µmol m^−2^ s^−1^); B (blue): 64 (µmol m^−2^ s^−1^). The plants were exposed to light for 16 h, the temperature was maintained at 21/19 °C (day/night), and RH was approximately 60–65%. The seeds were sown individually to rockwool fingers (25 × 25 × 40 mm, Grodan, ROCKWOOL B.V., Warsaw, Poland), which 48 h before were watered up with a standard nutrient solution. One tray had 180 pieces (10 × 18 individual fingers).

After placing the seeds in the tubes, they were covered with a layer of pearlite, and the temperature was maintained at 24 °C for the first 3 days. The emergence time for kohlrabi, radish, and spinach was 2–3 days, and for peas, about 5 days. The plants were placed in polyethylene (PE) containers (V 3.45 dm^3^), forming hydroponic stagnation.

### 4.2. Composition of the Medium

The plants were fertilised with a standard nutrient solution with the following chemical composition (mg/L) N-NH_4_ < 10, N-NO_3_ 150, P-PO_4_ 50, K 150, Ca 150, Mg 50, Mn 0.5, Zn 0.44, Cu 0.03, B 0.011; pH 5.50, EC 1.8 mS·cm^−1^. Two levels of iron nutrition (mg·dm^−3^) were tested: 1.5 and 3.0. The source of iron was Librel FeDP7 chelate (7% Fe; Royal Brinkman, Poznan, Poland). Additionally, the following fertilisers for hydroponic cultivation were used to prepare the medium: potassium nitrate (13% N-NO_3_, 38.2% K), calcium nitrate (14.7% N-NO_3_, 18.5% Ca), mono potassium phosphate (22.3% P, 28.2% K), potassium sulphate (44.8% K, 17% S), magnesium sulphate (9.9% Mg, 13% S), manganese sulphate (32.3% Mn), copper sulphate (25.6% Cu), borax (11.3% B) and sodium molybdate (39.6% Mo). Nitric acid (38%) was used to adjust the pH of the medium (at 5.5). The dose of nitrogen added in with the acid was taken into account in the balance of that component.

### 4.3. Measurement and Collection of Growth Parameter Data

Biometric measurements were taken at harvest, i.e., 14 days after the plants began to sprout. Harvest was conducted by cutting the plants a few millimeters above the fingers. To avoid order effects, no plants were collected from the edge rows. Plant height (shoot length, cm, using a ruler), as well as fresh and dry weight, were checked (g, parameters were measured using laboratory scales WTB200, R: 0.001 g; Radwag, Radom, Poland). All measurements were taken on 10 plants, randomly selected from each round. The dry matter was obtained by drying the plant material to constant weight at 105 °C for 24 h [55]. The dry matter ratio (%) was calculated with the following formula:

DM = (W_dry_/W_fr_) × 100,

where DM is the dry matter ratio (%), W_dry_ is the dry weight of the sample, and W_fr_ is the fresh weight of the sample.

The chlorophyll content index (CCI) was measured using the OSI CCM-200 Plus leaf chlorophyll meter (ADC BioScientific Ltd., Hoddesdon, UK). Chlorophyll fluorescence was measured using the OS1-FL modulated fluorometer (Optiscience, Hudson, NY, USA) half an hour after the termination of the period of exposure to light. The dark adaptation parameters were used to determine the maximum quantum yield of PS II (photosystem II): Fv/Fm = (Fm − Fo)/Fm (Fo—minimal fluorescence yield of the dark-adapted state, Fm—maximal fluorescence yield during the first saturation pulse after dark adaption) [56]. Both measurements were made a day before harvest.

### 4.4. Analysis of Mineral Content

All analyses were conducted on the aerial parts of the plants. The samples were dried for 48 h at 45–50 °C to a stable mass and then ground. Before mineralisation, the plant material was dried for 1 h at 105 °C. To analyse the total content of Fe, Mn, Zn, and Cu, the plant material (2.5 g) was dissolved in a mixture of concentrated nitric (ultra-pure) and perchloric acids (analytically pure) at a 3:1 ratio (30 cm^3^). After mineralisation, the following measurements were taken: Fe, Mn, Zn, and Cu—flame atomic absorption spectroscopy (FAAS) using the Carl Zeiss Jena 5 apparatus (Carl Zeiss Jena, Thornwood, NY, USA). The accuracy of the methods implemented to perform the chemical analyses and the precision of analytical measurements of the level of nutrients were tested by analysing the LGC7162 reference material (LGC standards), with an average nutrients recovery of 96% (N, P, K, Ca, Mg, Fe, Mn, Zn).

### 4.5. Experiment Design and Statistical Analysis

The study was conducted as a two-factor experiment in four replications in an independent system. One growth container, in which approximately 180 plants were grown, was treated as one replication. The results are the averages from four replications. The data were analysed using the ANOVA method. The differences between the means were estimated using Duncan’s test at a significance level of α = 0.05. The data was statistically analysed using Statistica 13.3 software (StatSoft Inc., Tulsa, OK, USA).

## 5. Conclusions

Currently, research on and development of functional food with a beneficial effect on human health and longevity are ongoing. This research highlighted that the biofortification of microgreens with an essential micronutrient, such as Fe, can be successfully performed in soilless cultivation. The ability to produce nutrient-rich, biofortified microgreens in a very short time (7–21 days), with the potential to exploit a wide variety of species, could contribute to the supplementation and diversification of the diet. Particularly valuable is the fact that they can also be grown in places with no access to daylight, increasing the application potential regardless of natural light conditions. With relatively low concentrations of Fe in the nutrient solution (1.5 and 3.0 mg/L), there was no significant effect of Fe on the fresh weight and length of the tested plants. Small differences, depending on the species, were noticed for the chlorophyll content index and fluorescence yield. The greatest effect of the applied doses of Fe was noticeable in the content of elements.

The species with the highest content of Fe was pea, although it was also not very susceptible to the increased iron level in the medium. Spinach turned out to be the most sensitive species to biofortification. Based on the above, when selecting species for biofortification, both the specifics of the species and the natural content of a given element in a plant should be taken into account. Even with low levels of Fe, there was antagonism between Fe and the other analysed micronutrients. Therefore, the form of administration and the dose of the element used for biofortification should be considered so that its increased quantity does not lead to a reduction in the content of other elements in the plant.

It can be assumed, considering the downward trend in biomass production, that with the increase in the concentration of iron, the potential stress of plants could increase. Moreover, the phenomena of ion antagonism between Fe and other metal micronutrients can occur. To sum up, in the soilless cultivation of microgreens, Fe biofortification at the level of 3.0 mg/L can be an effective tool for producing functional foods, especially on urban farms.

## Figures and Tables

**Table 1:** Effect of iron supplementation on the biomass yield of the studied species (g plant^−1^).

Treatment	Species (g·Plant^−1^)				Mean
	Kohlrabi	Pea	Radish	Spinach	
Fe 1.5	0.0675 e *	0.1438 a	0.1138 b	0.0813 d	0.1016 a
Fe 3.0	0.0538 f	0.1375 a	0.0988 c	c 0.0700 de	0.0900 b
Mean	c 0.0606 d	0.1406 a	0.1063 b	0.0756 c	

* Means with the same letter are not statically different (*p* ≤ 0.05). Separately for means.

**Table 2:** The content of microelements in microgreens (in mg kg^−1^dry matter), depending on the level of iron and species of plant.

Species	Treatment	Mineral Content (mg kg^−1^ dry Matter)			
		Cu	Fe	Mn	Zn
Kohlrabi	Fe 1.5	4.30 c *	109.40 d	72.80 b	26.65 c
	Fe 3.0	3.40 d	125.10 c	97.25 a	23.71 d
Pea	Fe 1.5	6.90 a	174.00 ab	27.45 d	62.95 a
	Fe 3.0	6.10 b	180.05 a	27.00 d	55.25 b
Radish	Fe 1.5	3.04 d	122.55 c	54.70 bc	24.49 cd
	Fe 3.0	2.25 e	123.55 c	62.70 b	22.25 d
Spinach	Fe 1.5	3.12 d	123.10 c	42.90 cd	22.40 d
	Fe 3.0	2.20 e	167.30 b	54.90 bc	21.70 d
Mean	Fe 1.5	4.34 a	132.26 b	49.46 b	34.10 a
	Fe 3.0	3.49 b	149.00 a	60.46 a	30.73 b

* Results in a column with the same letter are not statically different (*p* ≤ 0.05). Separately for means.

**Table 3:** The uptake of microelements in microgreens (in µg kg^−1^d.m.), depending on the level of iron and species of plant.

Treatment	Species (µg kg^−1^d.m)				Mean
	Kohlrabi	Pea	Radish	Spinach	
	Fe				
Fe 1.5	7.3463 e *	24.9875 a	14.0188 b	10.0675 d	14.1050 a
Fe 3.0	6.7375 e	24.7600 a	12.3950 bc	11.7500 cd	13.9106 a
Mean	7.0419 d	24.8738 a	13.2069 b	10.9088 c	
	Mn				
Fe 1.5	5.0738 b	3.9563 c	6.3375 a	3.5088 c	4.7191 a
Fe 3.0	5.2388 b	3.7138 c	6.2938 a	3.8575 c	4.7759 a
Mean	5.1563 b	3.8350 c	6.3156 a	3.6831 c	
	Zn				
Fe 1.5	1.7925 de	9.0313 a	2.7863 c	1.8313 de	3.8603 a
Fe 3.0	1.2775 e	7.6000 b	2.2325 cd	1.5238 de	3.1584 b
Mean	1.5350 c	8.3156 a	2.5094 b	1.6775 c	
	Cu				
Fe 1.5	0.2875 cd	0.9950 a	0.3500 c	0.2575 de	0.4725 a
Fe 3.0	0.1838 ef	0.8388 b	0.2275 def	0.1538 f	0.3509 b
Mean	0.2356 bc	0.9169 a	0.2888 b	0.2056 c	

* Results for individual microelements with the same letter are not statically different (*p* ≤ 0.05). Separately for means.

**Table 4:** r-Pearson’s coefficients for the correlation between variables.

	Yield	DM (%)	Length	SPAD	FV/FM	Content				Biomass	Uptake			
						Fe	Mn	Zn	Cu		Fe	Mn	Zn	Cu
Yield	1.00													
DM (%)	0.05	1.00												
Lenght	0.81	0.15	1.00											
SPAD	0.37	−0.37	0.43	1.00										
FV/FM	0.11	−0.07	−0.13	0.07	1.00									
Fe content	0.69	−0.10	0.81	0.70	0.03	1.00								
Mn content	−0.73	−0.01	−0.74	−0.51	−0.09	−0.62	1.00							
Zn content	0.67	0.24	0.95	0.41	−0.20	0.74	−0.67	1.00						
Cu content	0.50	0.29	0.85	0.29	−0.33	0.57	−0.48	0.93	1.00					
Biomass	0.80	0.37	0.79	0.23	0.12	0.56	−0.74	0.76	0.62	1.00				
Fe uptake	0.83	0.25	0.91	0.40	0.03	0.78	−0.76	0.86	0.72	0.94	1.00			
Mn uptake	−0.22	0.36	−0.42	−0.58	0.19	−0.47	0.57	−0.40	−0.31	−0.01	−0.20	1.00		
Zn uptake	0.75	0.29	0.94	0.39	−0.12	0.73	−0.72	0.96	0.86	0.89	0.96	−0.28	1.00	
Cu uptake	0.72	0.32	0.93	0.35	−0.16	0.68	−0.67	0.95	0.91	0.87	0.93	−0.23	0.99	1.00

## Data Availability

Not applicable.
